# Rare Breast Metastasis From Urothelial Carcinoma: A Case Report and Literature Review

**DOI:** 10.7759/cureus.85779

**Published:** 2025-06-11

**Authors:** Tarek Mohamed, Hamza Elhashamy, Mohamed Ramez, Adam Bickers, Jaspal Phull

**Affiliations:** 1 Urology, United Lincolnshire Hospitals NHS Trust, Lincoln, GBR; 2 Urology, Assiut University Hospitals, Assuit, EGY; 3 Urology, University of Texas MD Anderson Cancer Center, Houston, USA; 4 Histology, United Lincolnshire Hospitals NHS Trust, Lincoln, GBR

**Keywords:** breast cancer, metastasis, neoadjuvant therapy, palliative radiotherapy, urothelial cancer

## Abstract

Bladder cancer is a prevalent malignancy globally, significantly affecting patient quality of life, morbidity, mortality, and healthcare costs. Disparities in bladder cancer incidence and mortality rates among countries result from varying risk factors, detection methods, and diagnostic techniques. Enhanced imaging has led to more precise staging, while advancements in surgical techniques have been paired with superior chemotherapy protocols. Bladder cancer frequently metastasises to the lymph nodes, bone, liver, lung, and peritoneum, but rarely to the breast. Metastases to the breast originating from bladder cancer present difficulties in identification and require standard invasive techniques. We report a case of a 79-year-old female diagnosed with a muscle-invasive bladder tumour who underwent a positron emission tomography-computed tomography (PET-CT) scan that demonstrated significant localised activity within the right breast. An ultrasound scan and mammography of the right breast revealed a 10 mm ill-defined mass in the upper outer quadrant and two minor ill-defined masses measuring 6mm and 10mm in the upper inner quadrant, with no concomitant axillary lymphadenopathy. A core cut biopsy and immunohistochemistry of the prior lesions were performed, which are morphologically and immunophenotypically similar, supporting a diagnosis of metastatic urothelial carcinoma.

## Introduction

Bladder cancer is the ninth most commonly diagnosed cancer worldwide, with both incidence and mortality rates increasing as per the latest statistics published in 2022 [1]. Disparities in bladder cancer incidence and mortality rates among countries result from varying risk factors, detection methods, and diagnostic techniques.

Radical cystectomy is the conventional therapy for localised muscle-invasive bladder cancer and refractory high-risk non-muscle invasive bladder cancer not responding to Bacillus Calmette-Guérin (BCG) intravesical therapy [2,3]. Cisplatin-based neoadjuvant chemotherapy (NAC) has been employed since the 1980s and has lately shown a definitive survival advantage. It has emerged as a cornerstone of multimodal therapies for muscle-invasive bladder cancer [4,5].

Approximately 10% to 15% of patients diagnosed with bladder cancer are already metastatic at the time of diagnosis [6]. Prior to the advent of efficient treatment, individuals with metastatic bladder cancer had a median survival rate that seldom surpassed six months. Surgical excision of the original tumour constitutes a component of a multimodal strategy in diverse cancers, potentially enhancing survival rates and quality of life. Nonetheless, the function of surgery in metastatic bladder cancer remains undetermined [7].

The predominant sites for metastases originating from bladder cancer are lymph nodes in the pelvis, specifically the internal iliac, external iliac, and obturator lymph nodes, along with bone, liver, lung, and peritoneum. Various atypical sites have been documented, including the brain and skin [8], while breast metastases are exceedingly uncommon [9].

The predominant origin of a metastatic breast lesion is typically the contralateral breast, necessitating a comprehensive examination as an initial step upon the identification of a mammary lump. Therefore, prompt and precise detection of metastatic illness is essential for directing suitable treatment approaches.

## Case presentation

**Figure 1:**
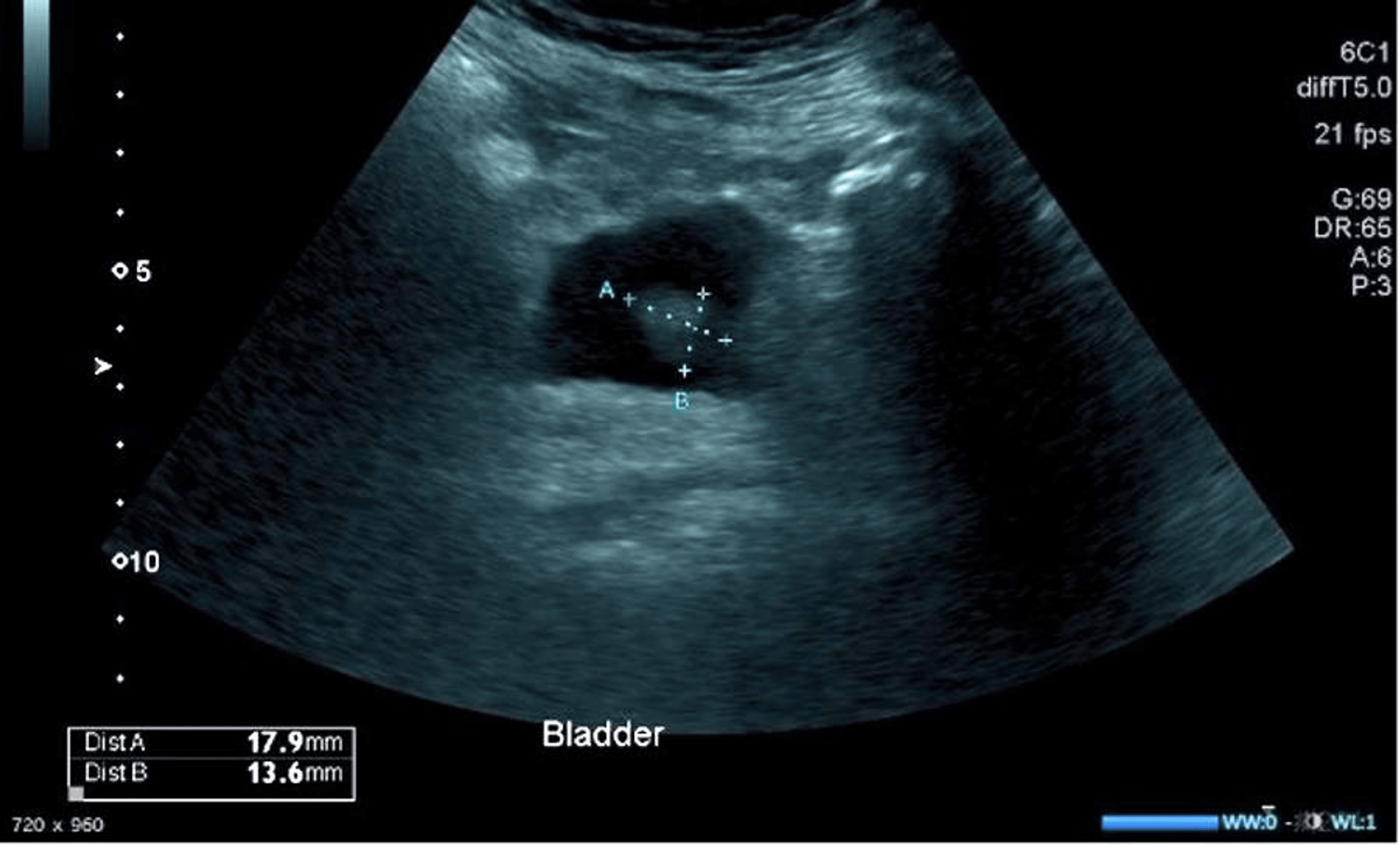
Ultrasound scan of bladder showed an 18x14mm hyperechoic bladder mass arising from the bladder wall.

A 79-year-old female patient was referred to a urology endoscopy clinic for visible haematuria associated with recurrent urinary tract infections. She underwent a flexible cystoscopy and ultrasound scan of the urinary tract, which confirmed the presence of an 18x14mm hyperechoic bladder mass arising from the bladder wall (Figure 1).

Therefore, a transurethral resection of the bladder tumour was performed, identifying a muscle-invasive, poorly differentiated and extensively necrotic transitional cell carcinoma (G3; at least cT2). A computed tomography (CT) scan of the chest and abdominopelvic showed a prominent right external iliac lymph node measuring 11mm in the short axis and a small nodule in the anterior upper segment of the right upper lobe of the lung measuring 5.5mm, and an indeterminate lumbar (L5) vertebral body change. She underwent an MRI of the spine, which confirmed small metastatic deposits in thoracic (T7), lumbar (L4), and L5 vertebral bodies, and there were no pathological fractures seen or definite extensions noted into the spinal canal.

**Figure 2:**
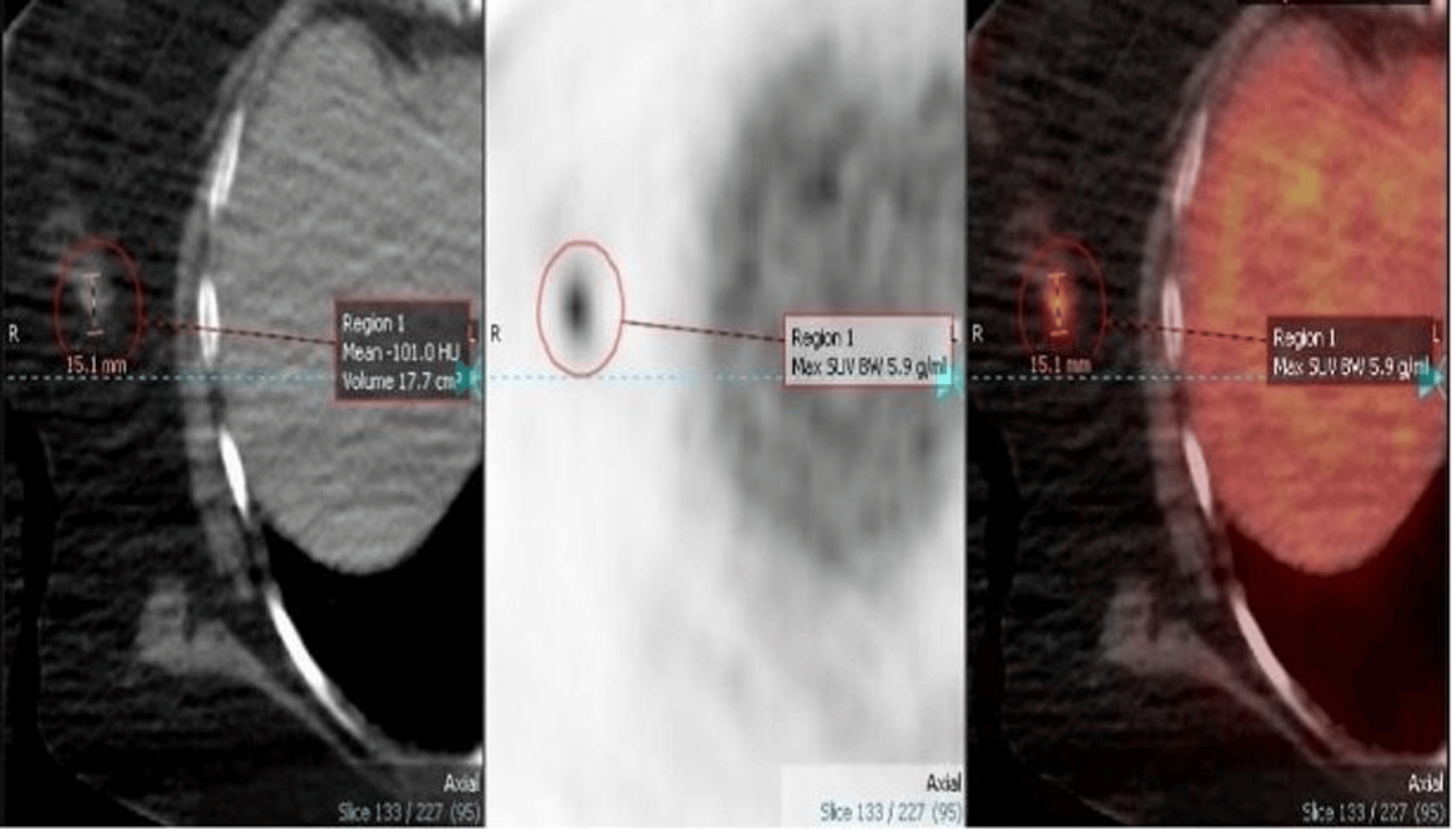
Whole-body FDG PET-CT showed an intense focal radiotracer uptake within the right breast. FDG: fluorodeoxyglucose; PET-CT: positron emission tomography-computed tomography

Correspondingly, the patient was investigated at another hospital for a clinical diagnosis of lung cancer and had a positron emission tomography-CT (PET-CT) scan, which revealed an intensely hypermetabolic cavitating right perihilar lung lesion, low-grade activity within a right low paratracheal node, and an intense focal activity within the right breast (Figure 2).

Subsequently, she underwent bronchoscopy washings for the right upper and middle lobes, which were negative for malignancy. She also underwent a biopsy, which revealed normal mucosa from the right middle lobe bronchus. The urology multidisciplinary consultation meeting decided to refer the patient to a breast multidisciplinary team (MDT) and proceed with histological evaluation of the specimen for programmed death-ligand 1 (PD-L1). 

She was seen in the breast surgery clinic and had a clinical examination of the right breast, which revealed slight nipple inversion, but there were no discrete lumps felt in the right breast. There was an area of glandular tissue in the upper outer quadrant, but no suspicious lumps were felt clinically, and the right axilla revealed no suspicious lymph nodes.

**Figure 3:**
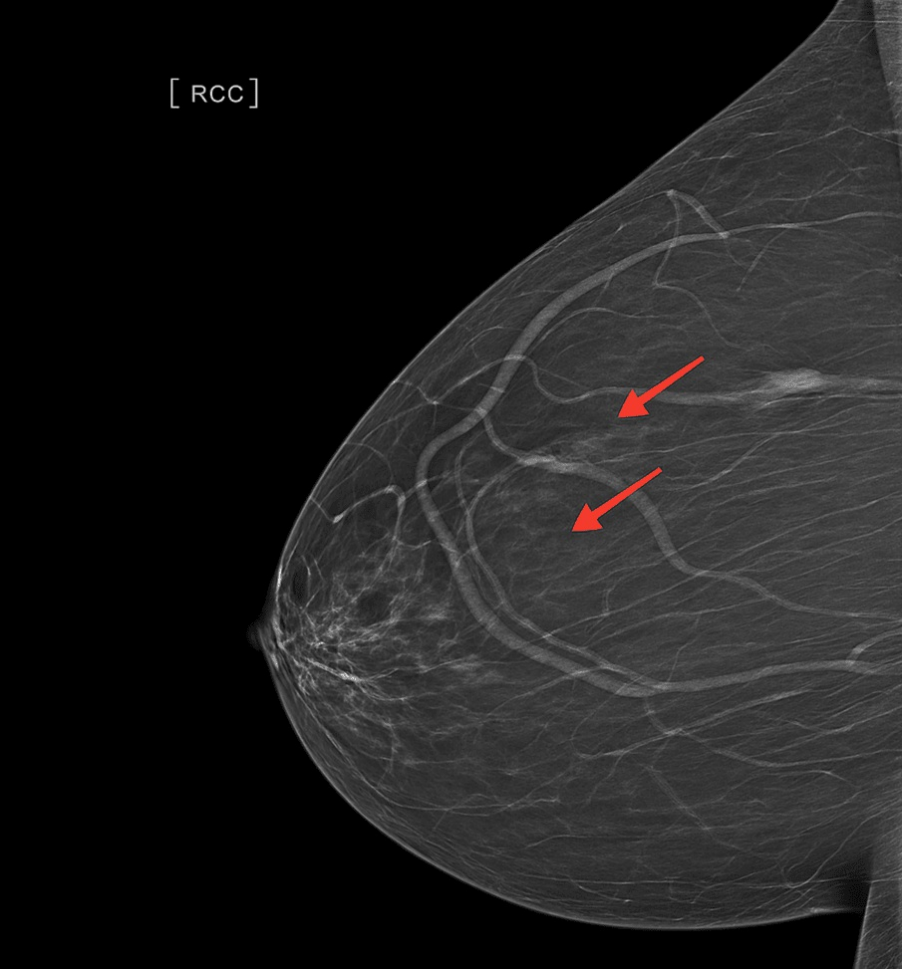
Mammogram of the right breast showed ill-defined mass, 6mm and 10mm, in the right upper inner quadrant (red arrows).

**Figure 4:**
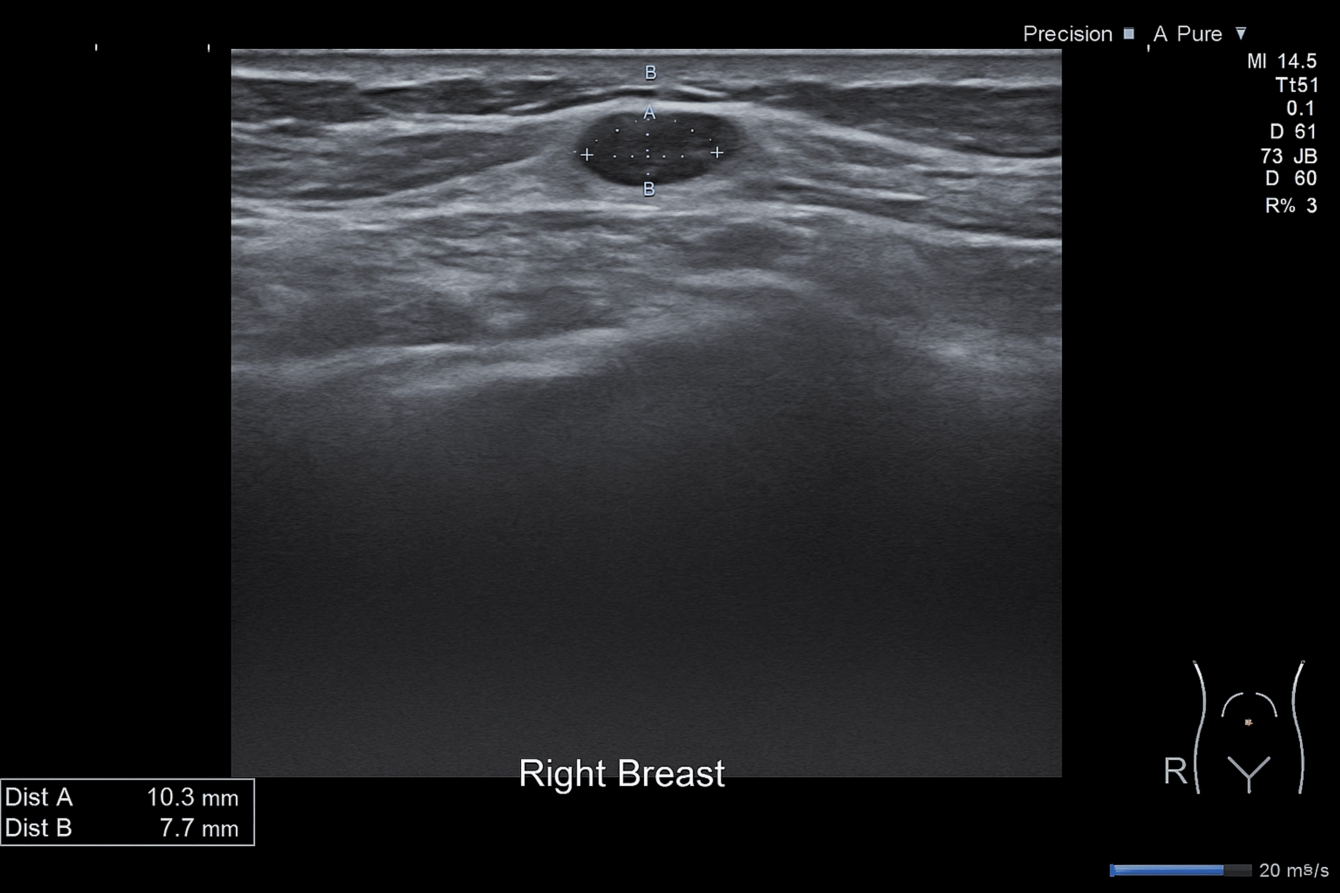
Ultrasound scan of the right breast showed a 10mm ill-defined mass in the upper inner quadrant.

An ultrasound and mammogram of her right breast confirmed a 10mm ill-defined mass in the upper outer quadrant. It also showed two small, ill-defined masses measuring 6mm and 10mm in the right upper inner quadrant, which were not associated with any axillary lymphadenopathies (Figures 3-4).

**Figure 5:**
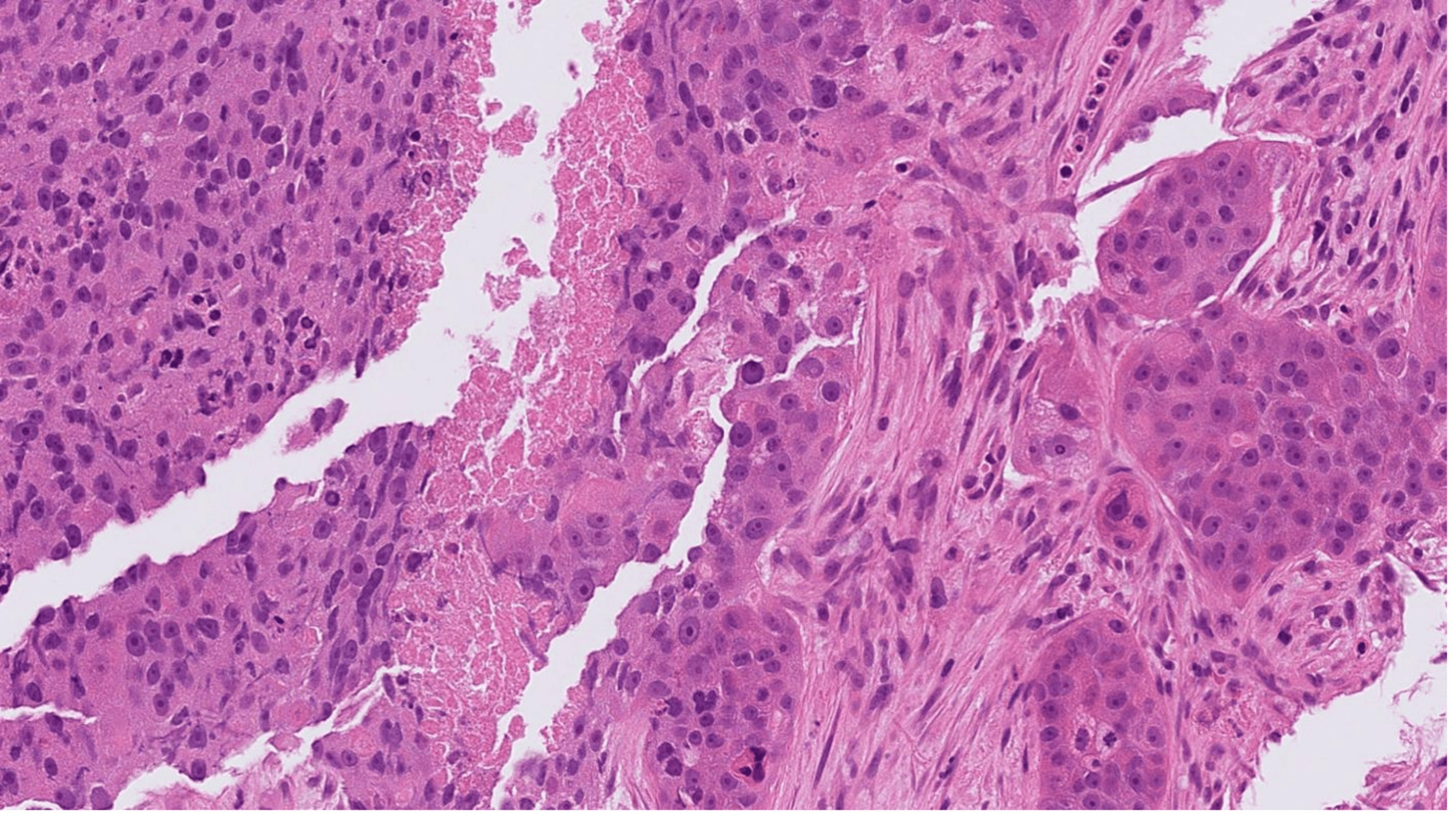
H&E stained sections at x20 magnification showing fibrofatty breast tissue infiltrated by poorly differentiated carcinoma. The morphology shows overlap between grade 3 invasive ductal carcinoma of NST and high-grade urothelial carcinoma. H&E: hematoxylin and eosin; NST: no special type

**Figure 6:**
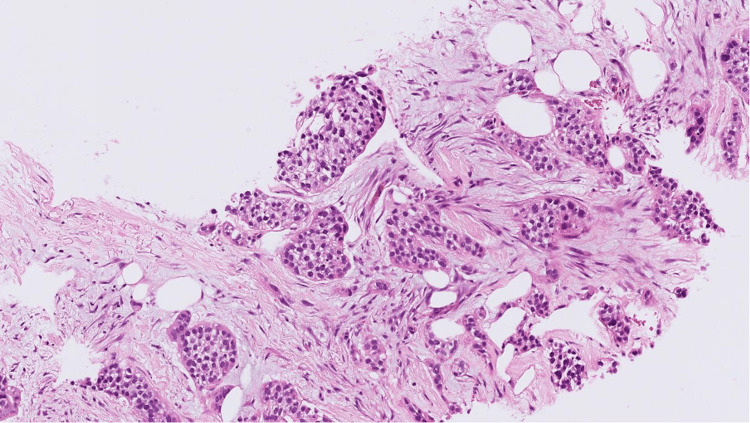
H&E stained sections at x10 magnification showing fibrofatty breast tissue infiltrated by poorly differentiated carcinoma. The morphology shows overlap between grade 3 invasive ductal carcinoma of NST and high-grade urothelial carcinoma. H&E: hematoxylin and eosin; NST: no special type

A core cut biopsy of the upper outer and inner masses was performed, which showed fibrofatty tissue infiltration by a high-grade carcinoma formed of pleomorphic epithelioid cells with focally clear cytoplasm in a desmoplastic stroma (Figures 5-6), and there was no normal glandular breast tissue or ductal carcinoma in situ found.

**Figure 7:**
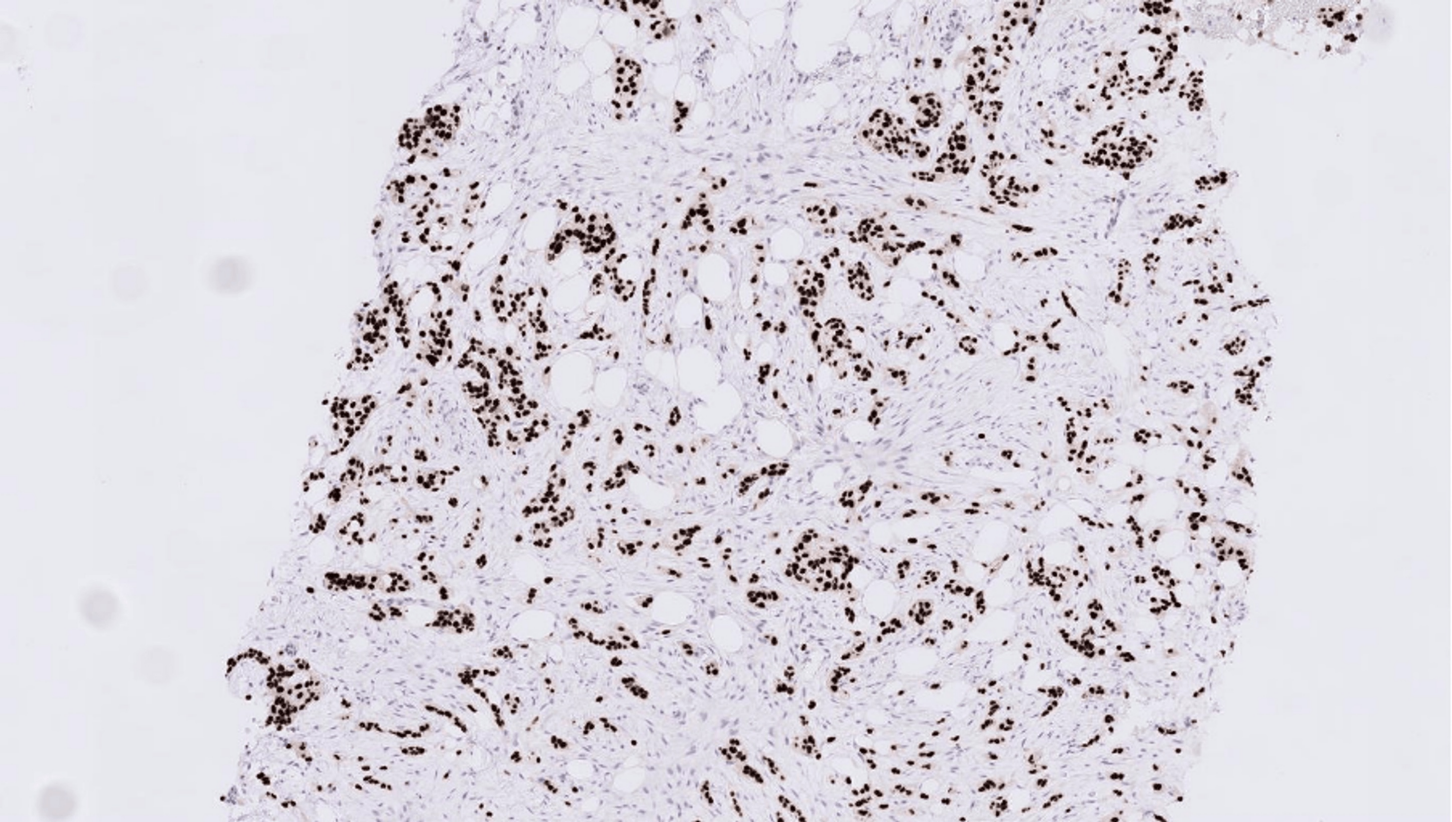
GATA3 immunohistochemistry showing strong nuclear staining in both the breast carcinoma and urothelial carcinoma.

**Figure 8:**
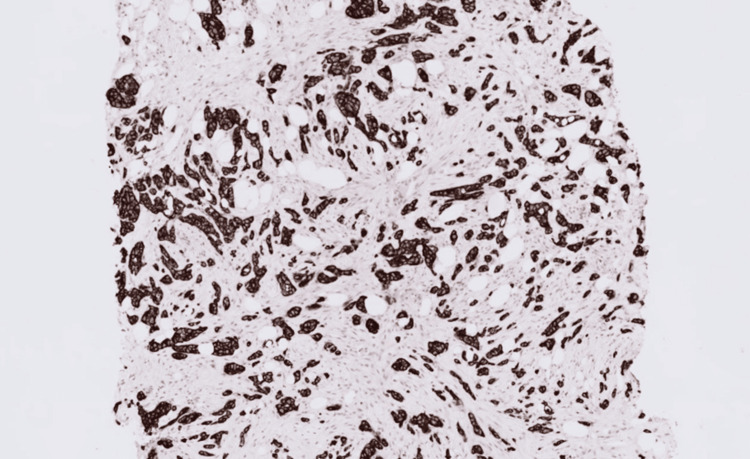
Cytokeratin 7 immunohistochemistry showing strong, diffuse cytoplasmic staining in both the breast carcinoma and urothelial carcinoma.

**Figure 9:**
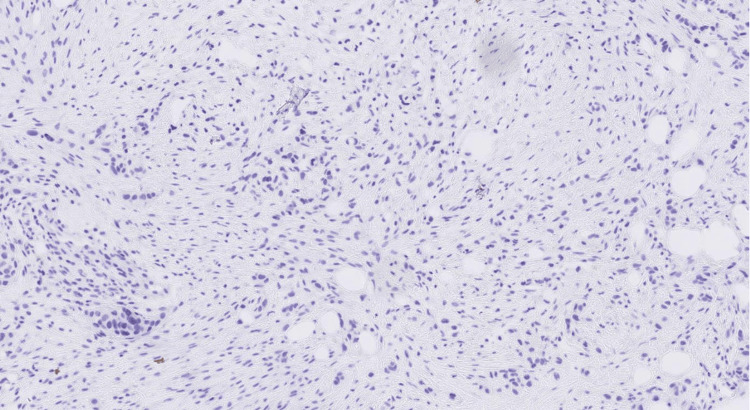
Immunohistochemistry showing negative staining for progesterone receptor.

**Figure 10:**
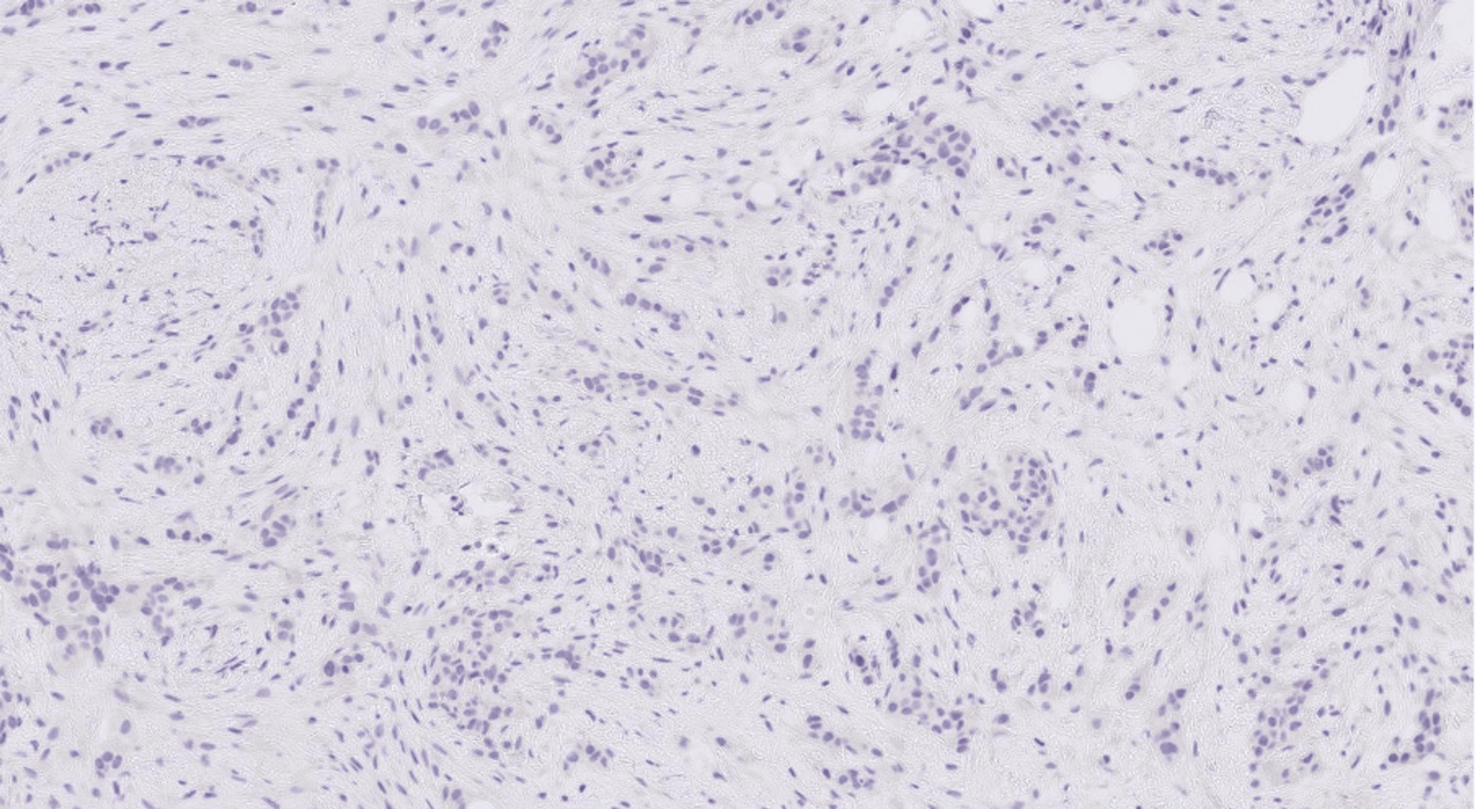
Immunohistochemistry showing negative staining for oestrogen receptor.

Immunohistochemistry has been performed and revealed that the carcinoma was strongly and diffusely positive for cytokeratin 7 (CK7), GATA-binding protein 3 (GATA3), and p40 (Figures 7-8), and was negative for oestrogen receptor (ER), progesterone receptor (PR), and paired box gene 8 (PAX8) (Figures 9-10).

**Figure 11:**
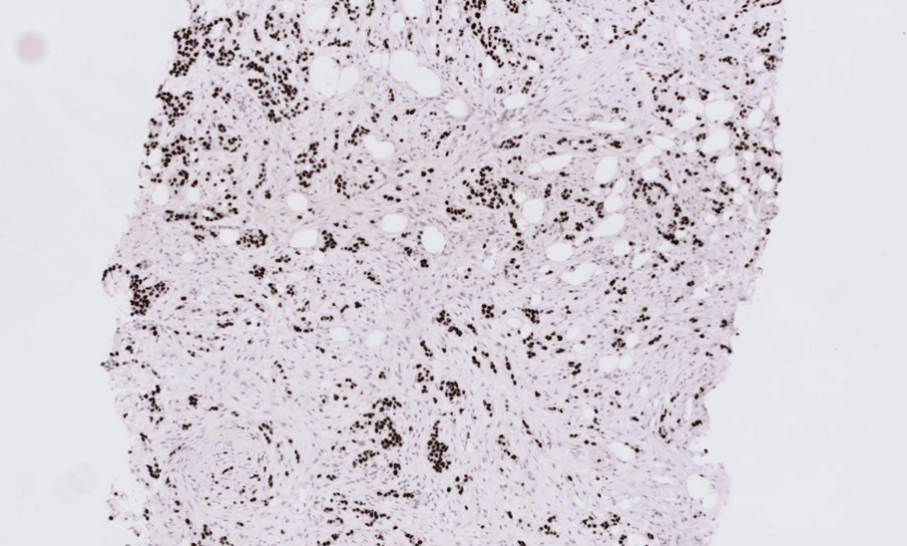
P40 immunohistochemistry shows strong, diffuse nuclear staining, which is more characteristic of urothelial carcinoma. Breast carcinoma typically does not express P40.

The immunohistochemistry profile of the bladder carcinoma was suggestive of a high-grade invasive urothelial carcinoma (Figure 11), which was compared with the previous breast lesion, and both the bladder tumour and the current breast tumour were morphologically and immunophenotypically similar, supporting a diagnosis of metastatic urothelial carcinoma.

The bladder tumour was tested with the Dako PD-L1 IHC 22C3 pharmDx test (Agilent Technologies, Santa Clara, CA, USA) to assess suitability for pembrolizumab therapy and Ventana PD-L1 SP142 assay (Ventana Medical Systems, Inc., Tucson, AZ, USA) to determine suitability for atezolizumab therapy, for which both tested negative for PD-L1 expression; hence, she is not eligible for immunotherapy. The patient was seen by consultant clinical oncologist who explained to her, despite having a performance status score of 1, how chemotherapy might not be in her best interest as this non-curative treatment would certainly compromise her quality of life, and its unlikely that any benefit in terms of prolonged survival would be linked with improved quality of life. The patient was subsequently treated with two weeks of pelvic palliative radiotherapy to control her pain. Unfortunately, she died after three months following the completion of palliative radiotherapy (six months from diagnosis).

## Discussion

The metastasis of bladder cancer to the breast represents an exceedingly rare phenomenon, posing a distinctive clinical challenge due to its atypical presentation and infrequent occurrence. While metastasis to distant sites is a well-documented aspect of cancer progression, the manifestation of bladder carcinoma in the breast remains an uncommon event, significantly deviating from the more conventional metastatic patterns observed in solid organ malignancies.

We searched PubMed for any reports of primary bladder cancer metastasis to the breast. Seven reports were identified. However, most of these reports did not report all the clinical and histological data in detail.

Lievore et al. reported a case of a 66-year-old female with a muscle-invasive high-grade urothelial carcinoma, with features of glandular and micropapillary differentiation, in which the PET-CT scan revealed increased uptake in the left iliac lymph nodes but no uptake in the breast. The ultrasound scan revealed a 7.8mm hypoechoic nodule. A biopsy of the nodule diagnosed a scarcely differentiated, invasive carcinoma with micropapillary characteristics that was suggestive of both breast primary tumour and bladder secondary lesion. An excisional biopsy of the mammary nodule was done and confirming the diagnosis of metastasis from micropapillary carcinoma of the bladder. The patient was treated with immunotherapy with pembrolizumab and demonstrated survival till the time of publication (10 months from diagnosis, six months from surgery) [9].

Another case was reported in a 59-year-old male patient who was diagnosed with high-grade urothelial carcinoma of the urinary bladder. During follow-up after chemotherapy, he presented with breast lumps on both sides, which were palpable and painful for two weeks, and skin redness on the left breast. Ultrasound-guided core needle biopsy was performed for the lesion in the left lower outer breast, and the histologic finding was metastatic carcinoma with plasmacytoid features from the urinary bladder [10].

Belton et al. reported another case of a 57-year-old female patient who presented with palpable subcutaneous lesions in the upper outer quadrant of the right breast 21 months after radical cystectomy and chemotherapy for high-grade invasive transitional cell carcinoma of the urinary bladder. The right breast lesions, along with a nodule on the patient’s back, were excised. Histologic studies of all lesions showed metastatic transitional cell carcinoma. The patient died eight months later despite aggressive chemotherapy [11].

Also, Wood et al. reviewed the findings in fine needle biopsy of extramammary malignancies presenting with breast metastases and found one case of transitional cell carcinoma of the bladder [12]. In addition, Silverman et al. reported the fine needle aspiration cytologic findings in 18 cases of metastatic neoplasms of the breast with one case of transitional-cell carcinoma of the urinary bladder [13].

Another study of 85 cases of non-mammary metastasis to the breast and axilla included two cases of bladder urothelial carcinoma [14].

Breast and bladder tumours may show morphological overlap, also in immunocytochemistry markers. CK7 is a cytokeratin commonly expressed in urothelial carcinoma, especially in high-grade tumours. GATA3 is a transcription factor expressed in breast and urothelial epithelial cells, and its positivity is a sensitive and specific marker for urothelial carcinoma. P40 is a marker for squamous differentiation, which can be seen in a subset of urothelial carcinomas [15]. Positivity for cytokeratin 20 also favoured the urothelial origin hypothesis, and negative oestrogen and progesterone receptors are widely described and show a poor prognosis [16].

Upon diagnosing breast metastasis from breast cancer, the preferred treatment is cisplatin-based chemotherapy. The role of surgery in metastatic urothelial carcinoma remains undefined, with the majority of insights derived from retrospective studies [7].

The clinical course of this histological variant is typically poor, with the five-year and 10-year overall survival rates in the largest study being 74 and 54%, respectively [17]. Furthermore, patients who have metastatic cancer from multiple sites to the breast have a poor overall prognosis [18]. This explains the rapid progression of the disease seen in our patient.

Understanding the implications of such an unusual metastatic route becomes imperative, given its potential influence on the prognosis, treatment strategies, and diagnostic intricacies involved in managing this distinct manifestation of cancer dissemination. Regrettably, the biological behaviour and potential prognostic factors of bladder cancer metastasis to the breast cannot be predicted based on the data from the reported cases.

## Conclusions

This case of bladder cancer spreading to the breast highlights the challenges in diagnosing and treating such rare metastases. The difficulties in differentiating these metastases from primary breast tumours emphasise the need for precise diagnostic tools. While treatments aimed at the original bladder cancer were used, addressing the breast metastases remains a challenge due to a lack of specific therapies. Patients with advanced bladder cancer and such metastases face uncertain outcomes, stressing the importance of continuous monitoring and further research to improve diagnosis and treatment options for these uncommon cases.
